# Case report: Transapical transcatheter double valve-in-valve replacement of degenerated aortic and mitral bioprosthetic valves with limited radiopaque landmarks

**DOI:** 10.3389/fcvm.2022.1086457

**Published:** 2022-12-13

**Authors:** Jiawei Zhou, Yuehuan Li, Haibo Zhang

**Affiliations:** Department of Cardiac Surgery, Beijing Anzhen Hospital, Capital Medical University, Beijing, China

**Keywords:** transapical, double valve-in-valve replacement, J-valve, TVI-transcatheter valve implantation, structural valve deterioration

## Abstract

A 67-year-old male patient who had undergone double valve replacement 11 years before presented with severe dyspnea to our department. The bioprosthetic aortic and mitral valves have failed. Because of the high risk of redo surgery. We perform a simultaneous transapical transcatheter valve-in-valve replacement of degenerated aortic and mitral bioprosthetic valves with limited radiopaque landmarks using the second-generation self-expanding J-valve. The post-operative course was stable and the patient was discharged on post-operative day eight.

## Introduction

With the increasing use of bioprosthetic valves, structural valve deterioration has become a major challenge for long-term prognosis. Valve-in-valve (VIV) is a minimally invasive and effective treatment for valve failure (1). VIV has been described in aortic, mitral, tricuspid and pulmonary positions, but it is usually performed on a single valve (2). Here we report a case of transapical double VIV replacement in a patient with severe mitral and aortic bioprosthetic valve regurgitation.

## Case presentation

The 67-year-old male patient had undergone double valve replacement for rheumatic valvular disease in 2011 with a 21-mm Medtronic Mosaic bioprosthesis (Medtronic, Inc, Minneapolis, MN, USA) in the aortic position and a 27-mm Medtronic Mosaic bioprosthesis (Medtronic, Inc, Minneapolis, MN, USA) in the mitral position. The patient underwent permanent pacemaker implantation due to a third-degree atrioventricular block. The patient was referred to our department recently, presenting with New York Heart Association (NYHA) grade IV dyspnea. Diuretic therapy is not effective. The patient also had a medical history of chronic lung disease and coronary atherosclerotic heart disease.

**FIGURE 1:**
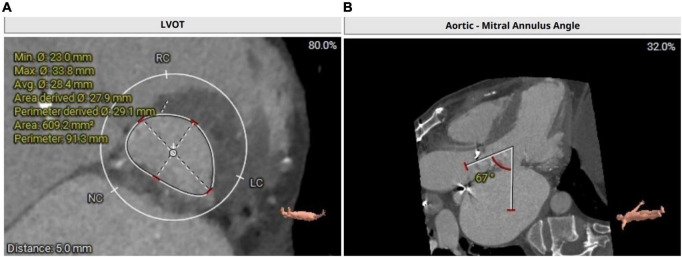
**(A)** Calculation of left ventricular outflow tract with an area of 609.2 mm^2^. **(B)** CT depicting a steep aorto-mitral annulus angle with 67°.

Transthoracic echocardiography revealed moderate bioprosthetic mitral valve stenosis (valvular orifice area of 1.8 cm^2^) with severe mitral valve regurgitation (regurgitant area of 16.2 cm^2^) and concurrent severe bioprosthetic aortic valve regurgitation, severe tricuspid regurgitation (regurgitant area of 18.8 cm^2^). Furthermore, the left ventricular end-diastolic diameter was 63 mm with a normal ejection fraction of 58%. Computed tomography angiography of the coronary artery showed a 50–60% stenosis in the middle segment of the anterior descending artery. The left ventricular outflow tract was calculated with 609 mm^2^ (Figure 1A). Aorto-mitral angle was steep with 67° (Figure 1B). Left coronary ostium height is 12.8 mm, right coronary ostium height is 14.3 mm. The frailty screening scale was four points. The preoperative logistic EuroSCORE II for redo surgery in this patient was calculated with 19.02%. Considering preoperative EuroSCORE II, the redo surgery and the extensive experience with transapical transcatheter aortic valve replacement (TAVR) procedures at our institution, the decision was made to perform a simultaneous transapical VIV procedure in the mitral and aortic positions.

**FIGURE 2:**
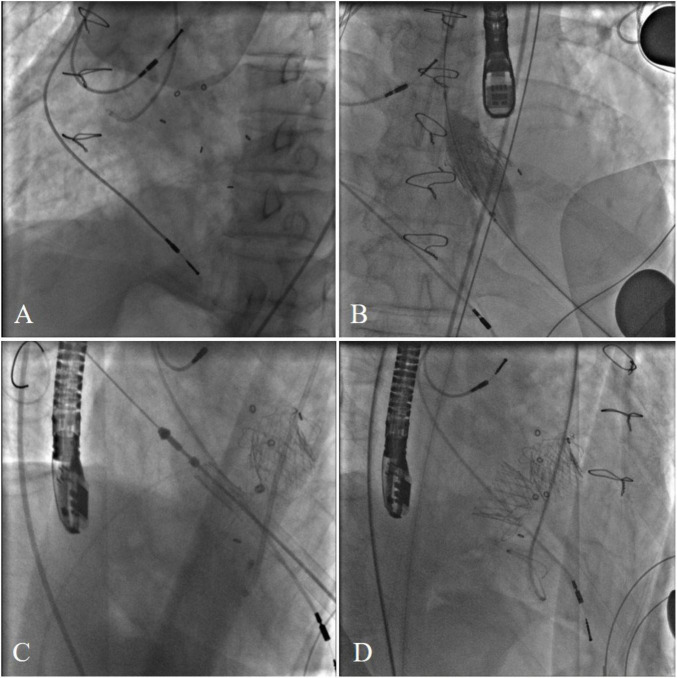
**(A)** The annulus of the Medtronic Mosaic aortic and mitral bioprosthesis was not visualized on fluoroscopy. **(B)** A total of 20 mm Atlas gold post-dilatation was used for post-dilatation in the aortic position. **(C)** Transcatheter bioprosthetic mitral valve implantation. **(D)** Final cardiac fluoroscopy showing both valves deployed and seated well.

The procedure was performed in a hybrid operating room. The chest was entered in the fifth intercostal space through a small left anterolateral incision. Two pledged purse-string sutures were placed at the apex. The annulus of the Medtronic Mosaic aortic and mitral bioprosthesis was not visualized on fluoroscopy (Figure 2A). After the apical puncture, a soft guide-wire and then a super stiff guide-wire were used to cross the bioprosthetic valve and into the aorta. The J-valve delivery device was inserted. Then, a 21-mm J-valve (Jiecheng Medical Technology, Suzhou, China) was deployed in the aortic position. Transesophageal echocardiography revealed massive perivalvular leakage. Therefore, a second 21-mm J-valve was implanted in the aortic position. A total of 20 mm Atlas gold post-dilatation was used for post-dilatation in the aortic position (Figure 2B). Thereafter the super stiff guidewire was placed in the left atrium through the mitral bioprosthetic valve. The J-valve was reversely loaded on the delivery system (Figure 2C). A 25-mm J-valve was deployed under rapid pacing in the mitral position. After that, a 25 mm Newman balloon was used for post-dilation. A post-operative fluoroscopic image with both aortic and mitral VIV replacements in place was taken (Figure 2D).

**FIGURE 3:**
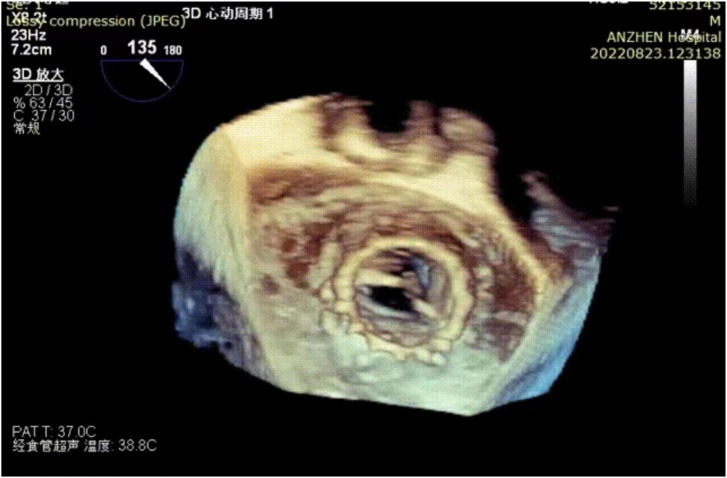
Three-dimensional transesophageal echocardiography reconstruction showing the mitral transcatheter heart valve.

Post-procedural echocardiography could detect neither paravalvular leakage nor aortic or mitral regurgitation. Three-dimensional transesophageal echocardiography showed a good shape of the mitral transcatheter heart valve (Figure 3). Echocardiography showing a mitral valve area of 1.7 cm^2^ and mitral valve mean gradient of 3 mm Hg and a transaortic gradient valve mean gradient of 13 mm Hg. The post-operative course was uneventful and without complication. The patient was discharged on post-operative day eight. After 2 months of follow-up, the patient’s NYHA class improved to grade 2.

## Discussion

Patients with bioprosthetic valves may contribute a higher incidence of subsequent repeat valve replacement in the future for structural deterioration. Although redo surgery is the current standard of care, this carries a significant risk of mortality (3). VIV could potentially be considered as viable alternatives in inoperable or high risks patients. Some patients may need underwent simultaneous double VIV procedures for the failed bioprosthetic valves. In this case, we present the simultaneous transcatheter transapical VIV for both failed bioprosthetic mitral and aortic valves with severe regurgitation using the J-Valve.

J-valve is a second-generation self-expanding transcatheter heart valve designed for transapical TAVR. It has been also proven effective and safe in transapical mitral VIV implantation (4). The transapical approach provides coaxial alignment and therefore reduces the risk of valve migration and left ventricular outflow tract obstruction. Bauernschmitt et al reported on the first transcatheter double valve replacement into native valves from transapical access (5). D’Onofrio et al believe that it should be considered the first access choice in these cases (2). However, Savoj et al reported one case of transcatheter double VIV replacement of the aortic and mitral bioprosthetic valve *via* the femoral artery (6).

There are several difficulties in this case. First, the double VIV at one puncture point of the heart apex, the spatial structure of the two biological valves may interact with each other. Second, the annulus of the patient’s original biological valve was not visualized on fluoroscopy. It can only be done under the guidance of three-dimensional ultrasound. Third, regurgitation was predominant in the aortic and mitral biological valves of the patient.

Regarding the order of deployment in double VIV, D’Onofrio et al suggested that aortic valve deployment should be done first in this procedure (2). There are several reasons for this order. There is an immediate afterload reduction and consequently better hemodynamic conditions for the mitral procedure after the aortic valve is implanted. Less risk of deployed mitral valve displacement or aortic valve malposition. In our case, we used the same sequence.

The VIV reports in the United States and Europe are mostly balloon-expandable valves (Sapien 3) (7). The Sapien 3 is anchored by radial support force, which is prone to displacement and paravalvular leakage after the operation. J-valve is a short stent valve, and its specific three locators design enables it to be firmly anchored in the failed valve. The three U-shape graspers of the J-valve are one-to-one buckled with the three bioprosthetic valve struts to avoid displacement to the left ventricle or left atrium. The self-expanding nitinol stent minimizes the risk of paravalvular leakage. The large left ventricular outflow tract area and short stent J-valve were used in this patient to reduce the risk of left ventricular outflow tract obstruction. Another advantage of J-valve is coronary protection. The positioning key of the J-valve can prevent the aortic bioprosthetic valve leaflet from getting closer to the coronary orifice and avoid coronary occlusion. In addition, the price of J-valve is much cheaper than Sapien 3.

Paravalvular leakage was significantly reduced after the second J-valve was released in the aortic position. The first J-valve did not fully expand after release. At the same time, due to the prior aortic bioprosthetic valves with limited radiopaque landmarks, the coaxiality of the released valve and the original biological valve is not ideal, resulting in paravalvular leakage. In this patient, we did not concurrently manage severe tricuspid regurgitation. We expected that the reduction in mitral regurgitation, combined with the use of diuretics, would reduce tricuspid regurgitation. Follow-up echocardiography did show a significant reduction in tricuspid regurgitation.

In conclusion, our case report shows the feasibility and efficacy of a double VIV procedure in the failed mitral and aortic bioprosthetic valve with a self-expanding valve *via* a transapical approach.

## Data availability statement

The original contributions presented in this study are included in the article/Supplementary material, further inquiries can be directed to the corresponding author.

## Ethics statement

Written informed consent was obtained from the individual(s) for the publication of any potentially identifiable images or data included in this article.

## Author contributions

JZ and YL contributed to composing the manuscript. JZ collected the patient’s data. YL and HZ revised the manuscript. All authors contributed to the article and approved the submitted version.
