# Supplementary figures and images for: Case report: Transapical transcatheter double valve-in-valve replacement of degenerated aortic and mitral bioprosthetic valves with limited radiopaque landmarks
Source: Front Cardiovasc Med. 2022 Dec 13;9:1086457. doi: 10.3389/fcvm.2022.1086457 (PMC9792843; doi:10.3389/fcvm.2022.1086457)

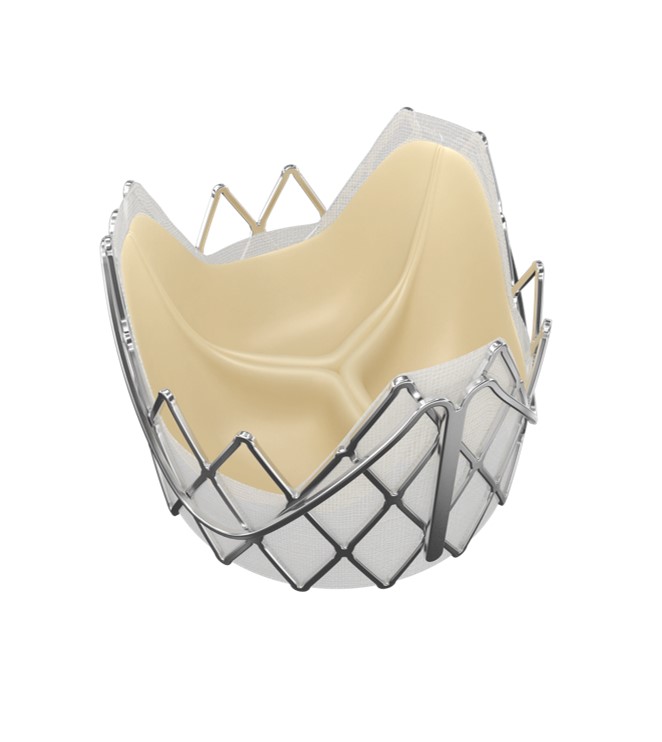


**J-valve**


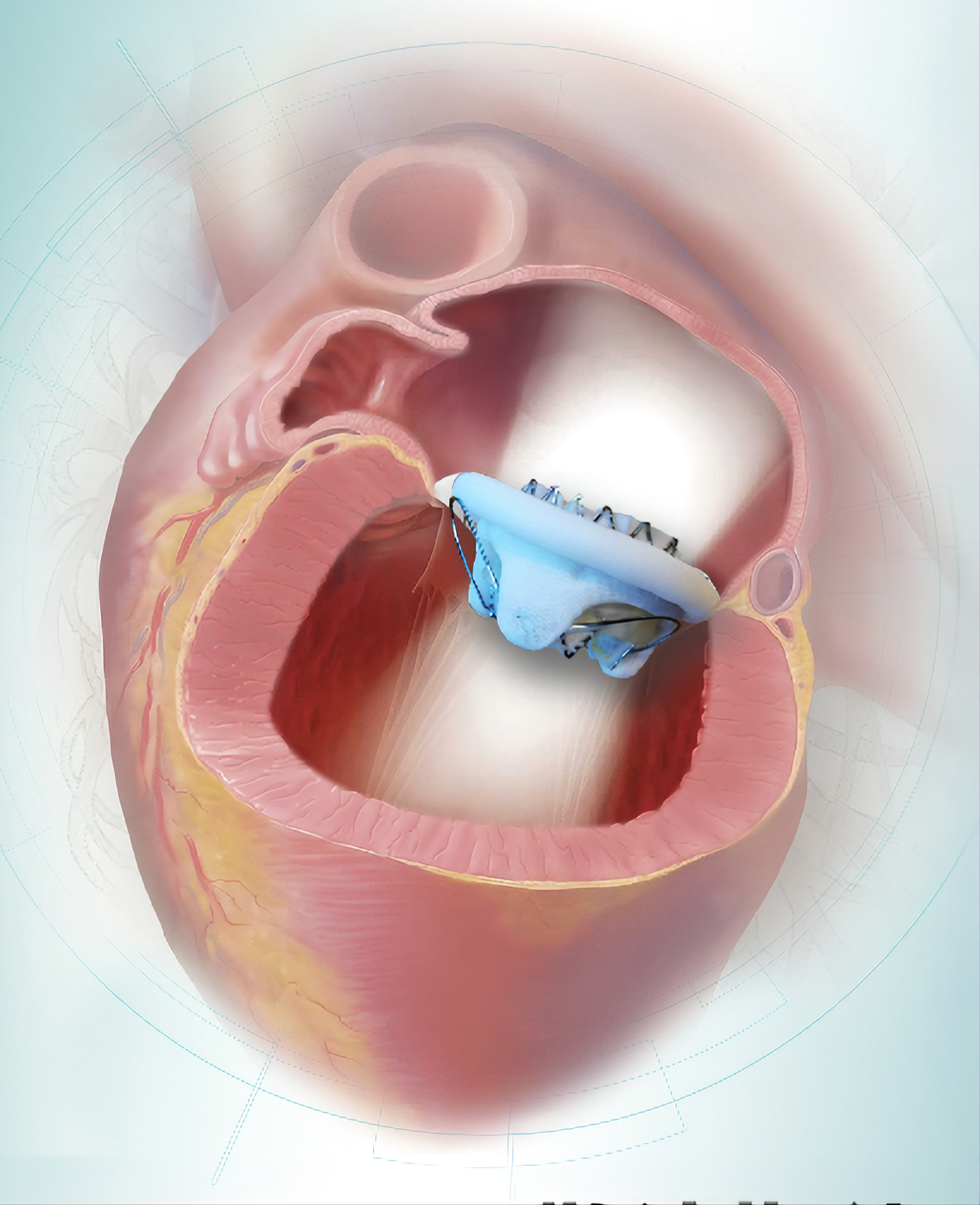


**Transapical Mitral VIV implantation with the J-valve.**

Supplement: Supplementary file 1 [file Table_1.DOCX]
